# The real‐world relationship between naltrexone/bupropion treatment and weight loss in Canada: A retrospective chart review

**DOI:** 10.1111/cob.12724

**Published:** 2024-12-01

**Authors:** Sean Wharton, Elham Kamran, Lehana Thabane, Peter Yin, Rebecca Christensen

**Affiliations:** ^1^ Wharton Medical Clinic Burlington Ontario Canada; ^2^ Faculty of Health Sciences McMaster University Hamilton Ontario Canada; ^3^ Toronto General Hospital University Health Network Toronto Ontario Canada; ^4^ Department of Health Research Methods, Evidence, and Impact McMaster University Hamilton Ontario Canada; ^5^ Research Institute of St Joe's Hamilton St Joseph's Healthcare Hamilton Hamilton Ontario Canada; ^6^ Bausch Health Quebec Canada; ^7^ Faculty of Kinesiology & Physical Education University of Toronto Toronto Ontario Canada

**Keywords:** bupropion, naltrexone, obesity, real‐world evidence, weight loss management

## Abstract

This study examined the change in weight at 6 months of naltrexone/bupropion (NB), a combination pharmacological therapy for weight management, in real‐world practice in Canada. The study was conducted through an observational, retrospective, single‐arm chart review of adult patients who attended the Wharton Medical Clinic in Ontario, Canada, between 2018 and 2021. The patients had a body mass index ≥30 or ≥27 kg/m^2^ with at least one weight‐related comorbidity. They were prescribed NB, titrated from 1 (8 mg/90 mg) to 4 tablets daily, along with lifestyle modification. Approximately 52% of 468 participants completed 6 months of treatment and 48.7% titrated to the maximum dose of 4 tablets daily. Participants were mainly female, with mean age of 49.5 years and BMI 38.4 kg/m^2^. After 6 months, participants lost a mean of 4.23 kg (95% confidence interval [CI] −4.99, −3.47) or 4.05% (CI −4.77, −3.34) of body weight, with 42.5% losing at least 5% of their body weight and 15.5% losing at least 10%. The most frequent adverse events were nausea (5.7%), constipation (5.7%), and headache (2.5%). Participants also experienced decreased appetite (14.7%), decreased cravings (13.9%), decreased hunger (9.4%) and felt full sooner (2.5%), which are changes likely to result in sustained healthy food choices and improved quality of life. The 6‐month NB treatment adjunct to lifestyle modification in a real‐world population resulted in clinically significant weight loss.

**What we already know about the subject**

- Behavioural treatments for managing weight in individuals with overweight and obesity have limited effectiveness due to challenges in maintaining adherence and weight loss, which are critical to mitigate associated health risks.
- Randomized controlled trials (RCT) of pharmacotherapies such as naltrexone/bupropion (NB) have shown that significant weight reduction could be achieved over 1 year of treatment.
- The effectiveness and safety of NB in real‐world populations remain, however, to be established.

**What this study adds**

- Individuals with overweight and obesity treated for 6 months with NB and adjunctive lifestyle modifications in a real‐world setting experienced significant weight loss.
- The weight loss was smaller than in the corresponding RCTs; however, NB showed a lower prevalence of adverse events, with nausea, constipation and headaches being the most reported.
- Behavioural changes, such as decrease cravings, decrease hunger and feeling full sooner, were also reported.

## 1 INTRODUCTION

Obesity is a serious chronic health condition that contributes to morbidity and mortality in Canada and worldwide.^1^, ^2^ Approximately 63% of Canadian adults are affected by overweight or obesity, with the obesity expected to rise among adults by at least 30% in the next two decades.^3^, ^4^ Obesity negatively affects quality of life and increases the risk of certain cancers, type 2 diabetes (T2DM), cardiovascular disease and other conditions.^1^, ^2^ While weight loss is recommended as a primary target to lower these risks,^2^, ^5^ the complex pathophysiology of obesity, which includes metabolic, hormonal and neurochemical adaptations that inhibit weight loss, makes losing weight a challenge.^1^

Behavioural treatments are the cornerstone of weight management, but the effectiveness of these treatments is limited by challenges of adherence and weight loss maintenance.^5^ Bariatric surgery can result in the greatest amount of weight loss among the available treatments, yet many patients are not willing or able to undergo this procedure.^6^, ^7^ Therefore, additional treatments, such as pharmacotherapies, are needed to improve the effectiveness of behavioural interventions. Pharmacological therapy leads to greater reductions in weight and better weight loss maintenance compared with lifestyle changes alone.^5^ In Canada, four therapies with clinically significant weight loss after at least 1 year are approved for the treatment of overweight and obesity.^8^ They consist of orlistat,^9^ liraglutide and semaglutide,^10^, ^11^ and the combination medication naltrexone/bupropion (NB).^12^ NB is an extended‐release, proprietary combination of naltrexone, an opioid receptor antagonist, and bupropion, an inhibitor of dopamine and norepinephrine reuptake. As an adjunct to lifestyle modifications, NB acts by modulating appetite via the hypothalamus and modulating signalling in the mesolimbic dopamine circuit to regulate reward pathways.^12^

Four randomized, placebo‐controlled clinical trials involving 4536 patients with overweight and obesity yielded 5.9%–11.5% weight loss among completers after 1 year of NB treatment.^13^, ^14^, ^15^, ^16^ In a network meta‐analysis of 168 trials, NB was also associated with reduced cardiovascular mortality and higher health‐related quality of life compared to placebo.^17^ However, the effectiveness and safety of NB have not been established in real‐world populations, which differ from those studied in randomized controlled trials (RCTs). This study aimed to examine the effectiveness of NB in a real‐world setting in Canada by describing changes in weight and adverse events (AEs) after 6 months of treatment and examining the association between demographic, prognostic factors and changes in weight.

## 2 MATERIALS AND METHODS

### 2.1 Study design

This study was an observational, single‐arm, retrospective chart review of patients who attended the Wharton Medical Clinic (WMC) in Ontario for obesity treatment between March 16, 2018 and May 18, 2021 and who were prescribed and had initiated NB as an adjunct to lifestyle modification. The WMC is a specialized weight and diabetes management clinic with multiple locations across southern Ontario, Canada, that operates under principles outlined in the Canadian clinical practice guideline for the treatment of overweight and obesity.^8^

Adults 18 years of age and over with a BMI ≥30 or ≥27 kg/m^2^ with at least one weight‐related comorbidity were included. Participants were required to have at least one self‐reported or device‐measured weight value during the 3 months prior to initiating NB, and to have initiated NB therapy during the selection period and attended at least one WMC visit during the follow‐up period. Patients who had previously undergone bariatric surgery, received NB therapy or were taking another medication for weight management were excluded.

The primary objectives of this study were (1) to determine the change in weight from baseline to 6 months and (2) to examine the association between baseline demographic/prognostic factors and changes in weight at 6 months. The secondary objectives included: describing changes in weight and cardiometabolic parameters at 6 months for the entire cohort and by obesity class; describing the percentage of patients achieving ≥5% and ≥10% weight loss at 4 and 6 months; examining the association between baseline demographic/prognostic factors and changes in weight at 4 months and discontinuation or end of follow‐up; and examining the distribution of time‐to‐maintenance dose, average maximum dose and persistence for the whole cohort and by obesity class. Changes in cardiometabolic parameters were investigated, including systolic and diastolic blood pressure (SBP, DBP), fasting blood glucose, high‐density lipoprotein cholesterol (HDL‐C), low density lipoprotein cholesterol (LDL‐C), haemoglobin A1C (HbA1C) and triglycerides.

The standard dosing schedule for NB begins with one 8 mg/90 mg tablet daily, increasing by one tablet per week until the maximum dose of four tablets daily (32 mg/360 mg) is reached at the start of week 4.^12^ At the WMC, a modified schedule is employed in which patients are started at one tablet daily and given the flexibility to increase the dose based on the recommend escalation schedule or on judgement of the physician. In accordance with common practice, patients were not asked to discontinue treatment after 16 weeks due to insufficient weight loss. Concurrent with NB, participants underwent lifestyle modifications that included increased physical activity and a reduced‐calorie diet that adhered to the Canadian Obesity Guidelines, which recommends healthy eating, reduced portion and caloric restriction.^18^ Caloric reductions were not calculated or recorded.

Weight measurements and other clinical assessments were performed at months 4 ± 1 and 6 ± 1 of treatment. Side‐effects and benefits of NB was self‐reported by the patient at each visit and recorded in their electronic medical record (EMR) by the medical doctor or the bariatric educator (during the COVID‐19 pandemic, some data were patient‐reported). Ethnicity of the participants was also self‐reported, on a voluntary basis. Patients provided informed consent for the use of their data for research purposes during their first visits to the WMC. The study was conducted on de‐identified EMR data in accordance with the Declaration of Helsinki and the Council for International Organizations of Medical Sciences (CIOMS) ethical guidelines, and the Good Pharmacoepidemiology Practices (GPP) guidelines for non‐interventional studies.^19^, ^20^, ^21^

### 2.2 Statistical analysis

Data were recorded as percentages for categorical variables and mean ± standard deviation (SD) or median (first quartile [Q1], third quartile [Q3]) for continuous variables. Multivariable linear regression was employed to determine the associations between continuous outcomes and baseline characteristics, in particular, age, sex and BMI or obesity class; binomial regression for binary outcomes; Kaplan–Meier plots for time‐to‐the‐outcome variables; as well as log‐rank statistics and Cox regression for distribution comparison between groups.

The estimated coefficient for linear regression, the risk ratio for binomial regression, as well as the corresponding 95% confidence intervals (CI) and associated *p*‐values, were reported. All multivariable regression models included patient age, sex and BMI. Alpha = .05 was used to determine significance, however, it was not adjusted for multiple testing since these analyses were exploratory.

Sensitivity analyses to explore the impact of missing data were performed for the primary endpoint using multiple imputation, which performs well under the assumption that data are missing at random.^22^, ^23^ The expectation–maximization algorithm was employed to generate 10 datasets for the multiple imputation analysis. This algorithm ‘computes the maximum likelihood estimates for parameters of multivariate normally distributed data with missing values’.^24^ All analyses were performed using SAS version 9.2 or 9.4 (Cary, NC, USA).

## 3 RESULTS

### 3.1 Patient disposition and baseline characteristics

The patient population (*n* = 468) was mainly female (*n* = 449; 95.9%) with a mean age of 49.5 (SD 12.2) years (Table 1). The mean BMI was 38.4 (SD 7.3) kg/m^2^ and the majority of patients had overweight or obesity either in class I (31.2%), II (29.3%) or III (33.1%) (Table 2). The mean HbA1C was 5.54 (SD 1.06) mmol/mol and the mean LDL‐C was 3.36 (SD 1.04) mmol/L (Table 2). Patients self‐reported 3.05 (SD 3.03) comorbidities on average (Table 1), hypertension being the most common (24.2%), followed by obstructive sleep apnoea (21.2%). Prediabetes was noted in 11.3% of patients and T2DM was seen in 4.3% of patients (Table S1). Anxiety and depression were common, each occurring in approximately 1 in 6 patients (Table S1).

**TABLE 1:** Patient demographics and baseline characteristics.

	Baseline
Individuals, *N*	486
Female, *n* (%)	449 (95.9)
Age in years, mean (SD)	49.5 (12.2)
*Race/Ethnicity*	
White/Caucasian, *n* (%)	130 (27.8)
South or East Asian, *n* (%)	7 (1.5)
Black, *n* (%)	4 (0.9)
Aboriginal, *n* (%)	1 (0.2)
Other, *n* (%)	12 (2.6)
Not known, *n* (%)	314 (67.1)
*Physiologic*	
Number of comorbidities, mean (SD)	3.05 (3.03)
Height in cm, mean (SD)	166.8 (9.2)
Waist circumference (cm), *n*, mean (SD)	169, 116.9 (15.5)

Abbreviations: *n*, individuals; SD, standard deviation.

**TABLE 2:** Outcomes at 4 and 6 months.

	Baseline		4 months		6 months		Estimated change at 6 months^a^		
	*n* (%)	Mean (SD)	*n* (%)	Mean (SD)	*n* (%)	Mean (SD)	*n*	Mean or odds^b^ (95% CI)	*p*‐value
Individuals, N	468 (100.0)	N/A	286 (61.1)	N/A	245 (52.4)	N/A	245	N/A	N/A
*Obesity class, n (%)*									
Overweight (BMI <30 kg/m^2^)	30 (6.4)	N/A	33 (11.5)	N/A	31 (12.7)	N/A	N/A	N/A	N/A
I (BMI 30–34.99 kg/m^2^)	146 (31.2)	N/A	89 (31.1)	N/A	86 (35.1)	N/A	N/A	N/A	N/A
II (BMI 35–39.99 kg/m^2^)	137 (29.3)	N/A	78 (27.3)	N/A	67 (27.5)	N/A	N/A	N/A	N/A
III (BMI ≥40 kg/m^2^)	155 (33.1)	N/A	86 (30.1)	N/A	61 (24.9)	N/A	N/A	N/A	N/A
*Physiologic parameters*									
Weight (kg)	468	107.2 (24.3)	286	103.3 (22.4)	245	101.6 (23.0)	245	−4.23 (−4.99, −3.47)	<.001
Weight change	N/A	N/A	286	−3.41 (4.87)	245	−4.23 (6.08)	N/A	N/A	N/A
Weight loss of ≥5%	N/A	N/A	90 (31.5)	N/A	104 (42.5)	N/A	245	0.74^b^ (0.57, 0.95)	.019
Weight loss of ≥10%	N/A	N/A	21 (7.4)	N/A	39 (15.5)	N/A	245	0.19^b^ (0.13, 0.27)	<.001
BMI (kg/m^2^)	468	38.4 (7.3)	286	37.4 (7.2)	245	36.6 (7.1)	N/A	N/A	N/A
*Cardiometabolic parameters*									
SBP (mmHg)	336	125 (12.4)	190	127 (14)	137	126 (11)	128	−0.64 (−2.48,1.20)	.497
DBP (mmHg)	336	77 (8.3)	190	78 (9.5)	137	77 (9)	128	0.27 (−1.30,1.85)	.735
Fasting blood glucose (mmol/L)	102	5.40 (0.71)	43	5.36 (0.63)	57	5.38 (0.96)	17	−0.15 (−0.32, 0.03)	.12
HDL (mmol/L)	110	1.36 (0.35)	48	1.41 (0.39)	59	1.40 (0.33)	20	0.05 (−0.03, 0.14)	.278
LDL (mmol/L)	93	3.36 (1.04)	41	3.14 (0.84)	47	3.44 (0.93)	13	−0.01 (−0.31, 0.29)	.957
HbA1C (mmol/mol)	112	5.54 (1.06)	48	5.61 (1.04)	59	5.56 (1.34)	20	−0.29 (−0.83, 0.25)	.303
Triglycerides (mmol/L)	110	1.60 (0.95)	48	1.43 (0.91)	59	1.46 (0.71)	N/A	N/A	N/A

Abbreviations: CI, confidence interval; *n*, individuals; N/A, not available; SD, standard deviation.

^a^ Unadjusted.

^b^ Odds.

### 3.2 Changes in weight and biomarkers

Of the 468 participants who entered the study, 286 (61.1%) remained at month 4 and 245 (52.4%) at month 6 (Table 2). The rest were lost to follow‐up. The discontinuation rate did not differ significantly by obesity class (log‐rank *p* = .151; Figure S1).

**FIGURE 1:**
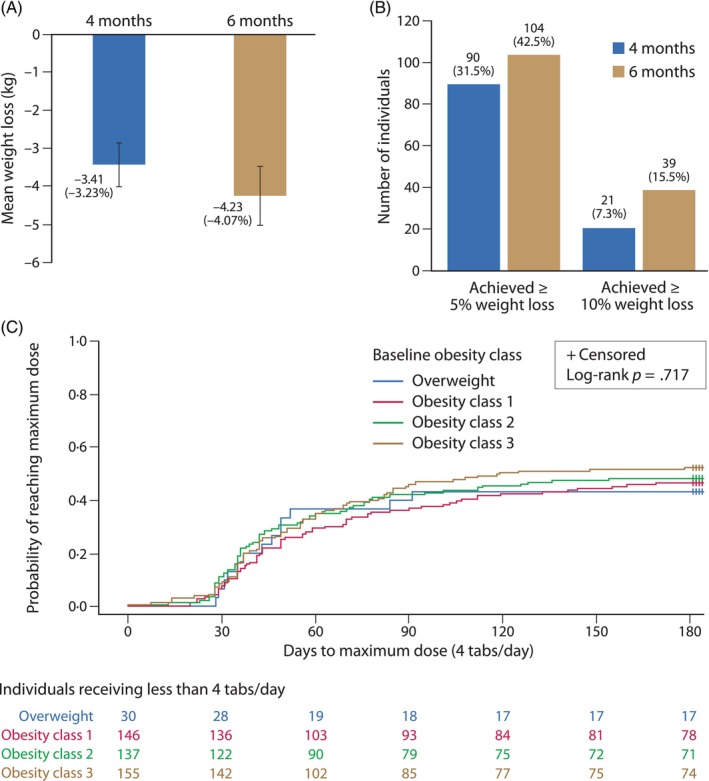
Weight loss achieved and maximum dose: (A) weight loss at months 4 and 6; (B) participants achieving at least 5% or 10% weight loss at months 4 and 6; (C) days to maximum dose (4 tablets/day) by obesity class.

Participants lost on average 3.41 (SD 4.87) kg, equivalent to 3.23% body weight (*p* < .001), by month 4, and 4.23 (SD 6.08) kg, equivalent to 4.07% body weight (*p* < .001), by month 6 (Figure 1A, Table 2 and Table S2). It was independent of the obesity class (Table S3). On average, participants' BMI decreased by 1.0 kg/m^2^ at month 4 and 1.8 kg/m^2^ at month 6 (Table 2).

At 6 months, 42.5% of patients on NB lost at least 5% of their body weight and 15.5% lost at least 10% of their body weight (Table 2 and Figure 1B). The odds ratios (OR) for these outcomes were 0.74 (CI 0.57, 0.95) for at least 5% weight loss and 0.19 (CI 0.13, 0.27) for at least 10% weight loss at 6 months, with lower odds ratios at 4 months (Table 2 and Table S2). Sensitivity analysis showed similar results (Table S4). There were no significant changes in cardiometabolic parameters and blood biomarkers by 6 months (Table 2).

### 3.3 Safety and behavioural outcomes

The most common adverse events reported were nausea (10.5% of participants at 4 months and 5.7% of participants at 6 months), constipation (6.3% and 5.7%, respectively) and headache (4.9% and 2.5%, respectively) (Table 3). Conversely, some participants reported positive effects of the medication other than weight loss such as decreased appetite (17.5% at 4 months and 14.7% at 6 months), decreased cravings (11.9% and 13.9%, respectively), decreased hunger (11.2% and 9.4%, respectively) or feeling full sooner (2.1% and 2.5%, respectively).

**TABLE 3:** Self‐reported symptoms at 4 and 6 months.

Symptom	4 months, *n* (%)	6 months, *n* (%)
Decreased appetite	50 (17.5)	36 (14.7)
Decreased cravings	34 (11.9)	34 (13.9)
Decreased hunger	32 (11.2)	23 (9.4)
Constipation	18 (6.3)	14 (5.7)
Nausea	30 (10.5)	14 (5.7)
Portion control	12 (4.2)	11 (4.5)
Headache	14 (4.9)	6 (2.5)
Feels full sooner	6 (2.1)	6 (2.5)
Dizziness	9 (3.2)	3 (1.2)
Insomnia/trouble sleeping	4 (1.4)	3 (1.2)
Diarrhoea	4 (1.4)	2 (0.8)
Anxiety	1 (0.4)	1 (0.4)
Dry mouth	4 (1.4)	1 (0.4)
Tinnitus	1 (0.4)	1 (0.4)
Vomiting	2 (0.7)	0 (0.0)
Hair loss	1 (0.4)	0 (0.0)
Increased satiety	1 (0.4)	0 (0.0)

*Note*: The symptoms are based on the review of the existing medical records at 4 and 6 months.

### 3.4 Dosage

About half of the participants (48.7%) reached the maximum dose of 4 tablets daily (Table 4) compared to 41.9% who remained at 1 or 2 tablets daily. However, 93% of the participants who completed the study were on the highest dose at the end of the study (Figure 1C). There was no apparent relationship between the mean highest dose and obesity class, with values ranging from 2.60 (SD 1.33) pills per day for participants with overweight to 2.89 (SD 1.29) pills per day for participants in obesity class III (Table S5). For the entire cohort, the mean highest dose was 2.84 (CI 2.72, 2.97) tablets per day (Table 4).

**TABLE 4:** Last and highest doses reached.

	*n* (%)	Mean (95% CI) or (SD)	*p*‐value
*Highest dose (tab/day)*			
1	127 (27.1)	N/A	N/A
2	69 (14.7)	N/A	N/A
3	44 (9.4)	N/A	N/A
4	228 (48.7)	N/A	N/A
*Estimates* ^a^			
Highest dose (tab/day)	468 (100)	2.84 (2.72, 2.97)	<.001
Last dose (tab/day)	468 (100)	2.65 (2.54, 2.77)	<.001
Time to last dose (day)	468 (100)	77.87 (56.51)	N/A
Time to last dose (weeks)	468 (100)	11.12 (8.07)	N/A

Abbreviations; CI, confidence interval; *n*, individuals; N/A, not applicable; SD, standard deviation.

^a^ Imputed values.

Overall, participants required 56.2 (SD 33.3) days or 8.1 (SD 4.8) weeks to reach the maximum dose (Table S5), longer than the recommended 28‐day dose escalation.^12^ Most participants (85%) who reached the maximum dose, did it between 30 and 90 days, regardless of their obesity class (log‐rank *p* = .717; Figure 1C). The time for titration to the maintenance dose (which could have been fewer than 4 tablets/day) was longer, at an average of 77.9 (SD 56.5) days or 11.1 (SD 8.1) weeks (Table 4).

## 4 DISCUSSION

While we observed a significant weight loss at 6 months, as reported in previous studies,^13^, ^14^ the 4% real‐world weight loss was lower than the 12‐month 5.9%–11.5% average loss reported in the NB randomized, placebo‐controlled, phase 3 trials: COR‐I, COR‐II, COR‐DM and COR‐BMOD.^13^, ^14^, ^15^, ^16^ In COR‐I, the largest NB trial, 870 participants who completed the study lost 8.1% of body weight, compared with 1.8% for placebo.^14^ In COR‐II, completers lost 7.8% of body weight on average at 28 weeks and 8.2% at 56 weeks, compared with 2.4% and 1.4%, respectively, in the placebo arm.^13^ In COR‐DM, the 505 participants with T2DM lost only 5.9% of body weight, compared with 2.2% in the placebo arm.^16^ Finally, in COR‐BMOD, which included additional intensive behavioural modification, participants lost 11.5% of their body weight, compared with 7.3% in the placebo arm.^15^ Similarly, 42.5% of participants in this study lost ≥5% of their body weight, less than the 48%–62% who did in COR‐I, COR‐II and COR‐BMOD at week 28.^13^, ^14^, ^15^

These results, demonstrating lower efficacy in the real world in comparison to RCTs, are consistent with real‐world setting where the population is more heterogeneous and adherence more variable.^25^ For example, this study included patients with T2DM (4.3%), CVD (5.3%) and non‐alcoholic fatty liver disease (15.4%), conditions that were absent in either COR‐I, COR‐II or COR‐BMOD.^13^, ^14^, ^15^ Participants in this study also had more hypertension (24.2%) compared with RCTs (e.g., COR‐I 19%–22%, COR‐II approximately 21%; patients with uncontrolled hypertension were excluded); were older (49.5 years compared with 44–46 years in RCTs), were more predominantly female (95.9% compared with 85%–92% in RCTs); and had a higher BMI (38 kg/m^2^ compared with 36–37 kg/m^2^ in RCTs).^13^, ^14^, ^15^

The 52% rate of completion in this study is consistent with the 50%–58% range observed in the NB trials,^13^, ^14^, ^15^ and a real‐world study of liraglutide at WMC where 52% to 57% of patients treated remained on pharmacotherapy at 6 months.^26^ In contrast, studies using behavioural interventions alone show high attrition rates,^27^ while a systematic review and network meta‐analysis of 28 RCTs comprising 29 018 participants treated with liraglutide, lorcaserin, NB, orlistat and phentermine‐topiramate indicated that completion rates could be as high as 70%.^28^ Trials for semaglutide showed completion rates between 80% and 92%,^29^, ^30^ with real‐world studies comparatively showing lower completion rates, ranging from 64% to 80%.^31^, ^32^ Discontinuations of NB could have been due to AEs, insufficient weight loss or cost considerations. Nonetheless, the results suggest that NB may be a feasible intervention in real‐world practice.

In our study, half of participants reached the recommended maximum dose of 32 mg/360 mg per day, which took almost 8 weeks, twice as long as the recommended 4 weeks.^12^ In the practice, patients had the flexibility to titrate up or down, as needed, which may have been influenced by factors such as the cost of the medication (insurance coverage for NB was limited during the study period), perceived effectiveness, and AEs.

The safety profile of NB in this study was consistent with RCTs, with nausea, constipation and headaches being the most frequently observed AEs.^13^, ^14^, ^15^ For example, in RCTs, headache occurred in 23.8% of patients in the NB arm and 17.5% of the placebo arm, and nausea in 34.1% of the NB arm and 10.5% of the placebo arm.^15^ In COR‐II, the corresponding percentages were 17.5% and 8.7% for headache and 29.2% and 6.9% for nausea.^13^ While these were the most frequent AEs in our study, they occurred at a lower frequency than in COR‐I and COR‐II, which may arise from differences in the ways AEs are collected and reported.

A limitation of this study is the ability to directly compare our results with data from RCTs due to differences in control over design parameters and data collection. For example, both the potential use of self‐reported weights—although considered reliable^33^—and the lack of adherence data could have affected the observed weight loss. More importantly, lower dosage likely contributed to a lower average weight loss as participants who only received 50% or less of the recommended dose did not stay on the medication for 6 months. Similarly, the lack of systematic collection of AEs and a lower average dosage possibly resulted in the lower frequency of AEs. Finally, the higher‐than‐expected participation of women in this study and the 52% completion rate may have introduced some clinical or socio‐economic biases that could increase the likelihood that a subset of the population either continues or discontinues the medication during the study period, potentially affecting weight loss outcomes. However, we do not expect these potential limitations to significantly affect the outcome of this study.

Our findings demonstrate that in a real‐world setting after 6 months of NB treatment with adjunctive lifestyle modification, participants lost 4% of their body weight, and over 40% lost 5% or more of their body weight, a clinically significant outcome. Patients also experienced decreased appetite, cravings, and hunger, which are changes likely to result in sustained healthy food choices and improved quality of life.

## FUNDING INFORMATION

Assistance with writing this manuscript was provided by STA Healthcare Communications, funded by Bausch Health Canada. L. T. received consulting fees from Bausch Health for the design, analysis and reporting of the data.

## CONFLICT OF INTEREST STATEMENT

S. W. received grants from Mitacs and the Canadian Institutes of Health Research (CIHR); received consulting fees, honoraria for academic lectures and writing support from Novo Nordisk, Boehringer Ingelheim, Eli Lilly and Bausch Health; received support for attending meeting from Novo Nordisk; participated on advisory boards for Novo Nordisk, Boehringer Ingelheim, Eli Lilly, Bausch Health and Biohaven; and occupied a role in Obesity Canada and the Obesity Society; all outside the submitted work. E. K. has nothing to disclose. L. T. received consulting fees from Bausch Health. P. Y. is an employee of Bausch Health. R. C. received consulting fees from Wharton Medical Clinic.

## Supporting information


**Data S1** Supporting Information

## Data Availability

De‐identified individual data that underlie the results presented in this article will be made available for the purpose of research, without limit of time. All requests to obtain the data should be directed to sean@whartonmedicalclinic.com
