# Supplementary material for: The real‐world relationship between naltrexone/bupropion treatment and weight loss in Canada: A retrospective chart review
Source: Clin Obes. 2024 Dec 1;15(2):e12724. doi: 10.1111/cob.12724 (PMC11907093; doi:10.1111/cob.12724)
Supplement: Supplementary file 1 — Data S1 Supporting Information [file COB-15-e12724-s001.pdf]

Supplementary Material

The Real-World Relationship Between Naltrexone/Bupropion Treatment and Weight Loss in Canada: A Retrospective Chart Review

Sean Wharton MD,[1,2,*] Elham Kamran MSc,[1,3] Lehana Thabane PhD,[4,5] Peter Yin MSc,[6] and Rebecca Christensen PhD[1,7]

[1] Wharton Medical Clinic, 2951 Walkers Line, Burlington, ON, Canada L7M 4Y1

[2] Faculty of Health Sciences, McMaster University, 1280 Main Street West, Hamilton, ON, Canada L8S 4L8

[3] Toronto General Hospital, University Health Network, 200 Elizabeth Street, Toronto, ON, Canada M5G 2C4

[4] Department of Health Research Methods, Evidence, and Impact, McMaster University, 1280 Main Street West, Hamilton, ON, Canada L8S 4L8

[5] Research Institute of St Joe's Hamilton, St Joseph's Healthcare Hamilton, 50 Charlton Avenue East,

Hamilton, ON, Canada L8N 4A6

[6] Bausch Health, 2150 St. Elzéar Blvd. West, Laval, QC, Canada H7L 4A8

[7] Faculty of Kinesiology & Physical Education, University of Toronto, 320 Huron Street, ON, Canada M5S 3J7

*** Corresponding author:** Sean Wharton MD, Wharton Medical Clinic, 2951 Walkers Line, Burlington, ON Canada L7M

4Y1; Tel: (833) 962-5276; Email: sean@whartonmedicalclinic.com

---

Contents

Table S1. Medical history of comorbidities at baseline. 2

Table S2. Additional outcomes at 4 and 6 months. 2

Table S3. Unadjusted and adjusted absolute weight change at 6 months. 3

Table S4. Sensitivity analysis of outcomes at 6 months. 4

Table S5. Highest dose and days to maximum dose. 5

Figure S1. Discontinuation rate by obesity class. 6

Table S1. Medical history of comorbidities at baseline.

Comorbidities	Baseline, n (%)
HYPERTENSION	113 (24.2)
OBSTRUCTIVE SLEEP APNEA	99 (21.2)
OSTEOARTHRITIS	90 (19.2)
DEPRESSION	86 (18.4)
GERD	84 (18.0)
HYPERCHOLESTEROLEMIA	78 (16.7)
ANXIETY	73 (15.6)
FATTY LIVER	72 (15.4)
HYPOTHYROIDISM	64 (13.7)
PREDIABETES	53 (11.3)
MUSCULOSKELETAL PAIN	45 (9.6)
IBS/IBD	39 (8.3)
CHOLECYSTECTOMY	35 (7.5)
ASTHMA	27 (5.8)
HEART DISEASE	25 (5.3)
DIABETES TYPE 2	20 (4.3)
PCOS	19 (4.1)
GALLSTONES	14 (3.0)
FIBROMYALGIA	10 (2.1)
THYROID DISEASE	10 (2.1)

GERD, gastroesophageal reflux disease; IBS/IBD, inflammatory bowel syndrome/inflammatory bowel disease; PCOS, polycystic ovary syndrome.

Table S2. Additional outcomes at 4 and 6 months.

	N	Estimate	p-value
Absolute change in weight (kg) 6 months-Baseline, mean (95% CI)†	468	-4.27 (-5.00,-3.55)	<0.001
Percent change in weight (%) 6 months-Baseline, mean (95% CI)†	468	-4.07 (-4.83,-3.31)	<0.001
Absolute change in weight (kg) 4 months-Baseline, mean (95% CI)‡	286	-3.41 (-3.98,-2.85)	<0.001
Percent change in weight (%) 4 months-Baseline, mean (95% CI)‡	286	-3.23 (-3.75,-2.71)	<0.001
Achieving weight loss of ≥5% at 4 months, Odds (95% CI)‡	286	0.46 (0.36,0.59)	<0.0001
Achieving weight loss of ≥10% at 4 months, Odds (95% CI)‡	286	0.08 (0.05,0.12)	<.0001

† Imputed values; ‡ No imputation. CI, confidence interval.

Table S3. Unadjusted and adjusted absolute weight change at 6 months.
Analysis performed without imputation on patients who completed the study (n=245).

Outcomes	Predictor Variables	Estimate (95% CI)	p-value
Absolute change in weight (kg) 6 months-Baseline [Mean Difference (95%CI)]	Unadjusted	-4.23(-4.99,-3.47)	<0.001
Absolute change in weight (kg) 6 months-Baseline [Coefficient (95%CI)]	Age (y)	-0.02(-0.09,0.04)	0.491
	Gender (f)	0.45(-3.26,4.15)	0.813
	Baseline BMI (kg/m^2)	-0.02(-0.14,0.09)	0.708
Absolute change in weight (kg) 6 months-Baseline [Coefficient (95%CI)]	Age (y)	-0.02(-0.09,0.04)	0.531
	Gender (f)	0.35(-3.36,4.06)	0.855
	Obesity Class (I vs 0)	-1.72(-5.74,2.29)	0.401
	(II vs 0)	-2.35(-6.4,1.69)	0.256
	(III vs 0)	-2.08(-6.12,1.96)	0.313

CI, confidence interval.

Table S4. Sensitivity analysis of outcomes at 6 months.

**Primary Outcomes – No Imputation**<br>Condition 1: Baseline weight must have been assessed on naltrexone/bupropion start date (n=428)<br>Condition 2: 6-month weight must be available (n=223)<br>Participants who satisfied both conditions (included in the analysis, n=223)<br>Participants who did not meet one or both conditions (excluded from the analysis, n=205)			
**Outcomes**	**Predictor Variables**	**Estimate (95% CI)**	**p-value**
Absolute change in weight (kg) 6 months-Baseline [Mean Difference (95% CI)] (n=223)	Unadjusted	-4.16(-4.95,-3.38)	<0.001
Absolute change in weight (kg) 6 months-Baseline [Coefficient (95% CI)] (n=223)	Age (y)	-0.01(-0.08,0.05)	0.680
	Sex (f)	0.67(-2.98,4.31)	0.721
	Baseline BMI (kg/m^2)	0.03(-0.09,0.15)	0.589
Absolute change in weight (kg) 6 months-Baseline [Coefficient (95% CI)] (n=223)	Age (y)	-0.01(-0.08,0.06)	0.730
	Sex (f)	0.37(-3.28,4.02)	0.843
	Obesity Class (I vs overweight)	-1.68(-5.65,2.28)	0.406
	(II vs overweight)	-2.56(-6.55,1.44)	0.211
	(III vs overweight)	-1.71(-5.7,2.27)	0.400
Percent change in weight (%) 6 months-Baseline [Coefficient (95% CI)] (n=223)	Unadjusted	-4.03(-4.78,-3.28)	<0.001
Percent change in weight (%) 6 months-Baseline [Coefficient (95% CI)] (n=223)	Age (y)	-0.01(-0.08,0.05)	0.662
	Sex (f)	0.31(-3.16,3.77)	0.862
	Baseline BMI (kg/m^2)	0.09(-0.02,0.2)	0.127
Percent change in weight (%) from baseline to 6 months [Coefficient (95% CI)] (n=223)	Age (y)	-0.01(-0.08,0.05)	0.725
	Sex (f)	-0.09(-3.57,3.39)	0.961
	Obesity Class (I vs overweight)	-1.62(-5.39,2.16)	0.403
	(II vs overweight)	-1.96(-5.76,1.85)	0.315
	(III vs overweight)	-0.66(-4.46,3.15)	0.736
**Primary Outcomes with Multiple Imputation†**<br>Condition 1: Baseline weight must have been assessed on naltrexone/bupropion start date (n=428)<br>Condition 2: 6-month weight must be available<br>Of the 428 Participants, 223 have weight assessed at 6 months<br>Of the 428 participants, 205 did not have weight assessed at 6 months<br>6-month weight imputed for 205 participants			
Absolute change in weight (kg) 6 months-Baseline [Mean	Unadjusted	-4.31(-5.03,-3.59)	<0.001

Difference (95% CI)] (n=554)			
Absolute change in weight (kg) 6 months-Baseline [Coefficient (95% CI)] (n=554)	Age (y)	-0.01(-0.08,0.06)	0.746
	Sex (f)	1.26(-2.02,4.43)	0.450
	Baseline BMI (kg/m^2)	0.06(-0.07,0.18)	0.375
Absolute change in weight (kg) 6 months-Baseline [Coefficient (95% CI)] (n=554)	Age (y)	-0.01(-0.08,0.05)	0.712
	Sex (f)	1.06(-2.23,4.36)	0.524
	Obesity Class (I vs overweight)	-0.53(-3.57,2.51)	0.728
	(II vs overweight)	-0.74(-4.11,2.63)	0.662
	(III vs overweight)	-0.08(-3.43,3.27)	0.962
Percent change in weight (%) 6 months-Baseline [Coefficient (95% CI)] (n=554)	Unadjusted	-4.17(-4.84,-3.50)	<0.001
Percent change in weight (%) 6 months-Baseline [Coefficient (95% CI)] (n=554)	Age (y)	-0.01(-0.07,0.05)	0.753
	Sex (f)	0.68(-2.39,3.74)	0.664
	Baseline BMI (kg/m^2)	0.12(0.02,0.22)	0.024
Percent change in weight (%) from baseline to 6 months [Coefficient (95% CI)] (n=554)	Age (y)	-0.01(-0.08,0.05)	0.695
	Sex (f)	0.43(-2.65,3.50)	0.785
	Obesity Class (I vs overweight)	0.22(-3.15,3.60)	0.894
	(II vs overweight)	0.56(-3.18,4.30)	0.763
	(III vs overweight)	1.76(-1.76,5.28)	0.317

† MI analysis: Number of imputed datasets 10. Baseline variables used to generate the imputed datasets: age, weight, height, sex, BMI.
CI, confidence interval.

Table S5. Highest dose and days to maximum dose.

Baseline Obesity Class	Highest dose, mean (SD)	Received 4 pills, n	Days to Dose of 4 Pills, mean (SD)
Overweight	2.60 (1.33)	13	46.62 (19.92)
I	2.80 (1.28)	68	62.07 (37.69)
II	2.77 (1.34)	66	52.35 (31.46)
III	2.89 (1.29)	81	55.83 (32.24)
Total	2.81 (1.30)	228	56.16 (33.32)
Never received 4 pills	n/a	240	n/a

n, individuals; n/a, not applicable; SD, standard deviation.

Figure S1. Discontinuation rate by obesity class.
Discontinuation is measured as the number of days to last dose.

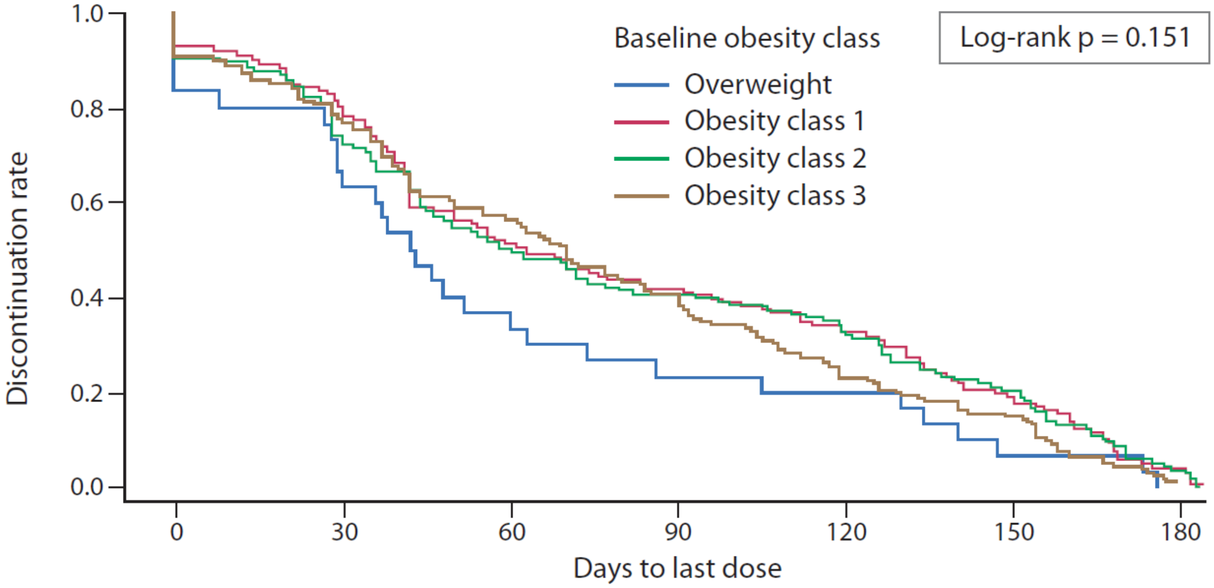

	0	30	60	90	120	150	180
Overweight	30	20	11	7	6	2	0
Obesity class 1	146	117	75	61	48	28	6
Obesity class 2	137	101	69	56	45	28	6
Obesity class 3	155	120	87	63	36	23	2
